# Overcoming the Double Empathy Problem Within Pairs of Autistic and Non-autistic Adults Through the Contemplation of Serious Literature

**DOI:** 10.3389/fpsyg.2021.708375

**Published:** 2021-07-27

**Authors:** Melissa Chapple, Philip Davis, Josie Billington, Joe Anthony Myrick, Cassie Ruddock, Rhiannon Corcoran

**Affiliations:** ^1^Department of Primary Care and Mental Health, School of Psychology, University of Liverpool, Liverpool, United Kingdom; ^2^Centre for Research Into Reading, Literature and Society, University of Liverpool, Liverpool, United Kingdom; ^3^Independent Researcher, Hamtramck, MI, United States; ^4^Independent Researcher, Gloucester, United Kingdom

**Keywords:** autism, autistic community, literary fiction, neurodiversity, emotional intelligence, double empathy

## Abstract

Recent research based on the needs of the autistic community has explored the frequent social misunderstandings that arise between autistic and non-autistic people, known as the double empathy problem. Double empathy understandings require both groups to respect neurodiversity by focussing on individuality across groups. This study aimed to explore how literature, through its ability to uncover nuanced emotional response differences between readers, could facilitate double empathy understandings within pairs of autistic and non-autistic adults. A longitudinal, qualitative design was used, with 4 gender-matched pairs. Participants read *Of Mice and Men* for 1 week, whilst completing a structured, reflective diary. This was followed by 4 one-hour paired reading sessions, where pairs discussed the book and their reflections in depth. Participants were then invited to a final one-on-one interview to discuss their thoughts and experiences of the paired reading sessions. Thematic and literary analysis of the session and interview data revealed four themes (1) The Book as Social Oil; (2) From a World of Difference to a World of Affinity; (3) Emotional Intelligence: From Thinking About to Feeling with; and (4) From Overwhelming to Overcoming. All participants reported having achieved an individualised view of one another to explore their nuanced differences. The non-autistic group reported a more sensitive understanding of what it means to be autistic, while the autistic group overcame concerns about non-autistic people stereotyping autism, and instead reported feeling valued and accommodated by their non-autistic partners.

## Introduction

Autism is a neurodevelopmental condition that results in distinguishably different socio-cognitive processing styles which pose advantages and disadvantages within current societal norms (Fletcher-Watson and Happé, 2019; Robinson et al., 2019). Since the identification of autism as a condition in the 1940s, the framing of autistic people has been dominated by the medical model of disability (Waltz, 2013). More recently, self-identification as autistic has become an important route to inclusion within the autistic community, promoting belongingness and improved self-understanding (Lewis, 2016). However, with many individuals continuing to rely on medical diagnosis for identification (Mogensen and Mason, 2015; Leedham et al., 2020), the medical model continues to influence how autism is thought about and explored, resulting in deficit-based conceptualisations and priorities (Waltz, 2013; Kapp, 2020). These deficit-based approaches result in a “lock and key” mentality toward autistic individuals, assuming that they need to be unlocked in some way to bring their information processing style closer to typical human neurocognition (Waltz, 2013). The problem with this approach is that it rests on the assumption that there is a typical form of human neurocognition, a state of “neuronormativity” often referred to as being neurotypical (Milton, 2020; Mueller, 2020).

As a result of these assumptions, dominant theories such as the mindblindness, empathising-systemising, and extreme male brain (Baron-Cohen, 1997, 2002, 2009) theories have viewed and explained autism through a largely deficit-based lens. These theories build upon a key underpinning idea that autistic individuals have profound perspective-taking difficulties, otherwise known as theory of mind deficits (Baron-Cohen, 1997). This long-standing assumption has led to a belief that autistic individuals have fundamentally impaired social abilities (Baron-Cohen, 2009; Lombardo and Baron-Cohen, 2011). Furthermore, there is an embedded assumption of impaired emotional intelligence amongst autistic individuals, with assumed deficits in recognising and empathically responding to the emotions of others (Baron-Cohen, 2009; Bodner et al., 2015; Rigby et al., 2018). From these theoretical assumptions and medicalised framings, intervention research has typically sought to alter the differential socio-cognitive processing styles that result from being autistic (Waltz, 2013; Pearson and Rose, 2021). In this way, it is seen as advantageous to bring the behaviours of autistic people closer to those associated with neurotypicality (Waltz, 2013). However, any consequent behavioural changes are thought, by some, to be short-term and brought about by conformity pressures (Mueller, 2020).

In contrast, social models of disability oppose these deficit-based assumptions. Instead, social models explore disability that results from disadvantages bounded in social construction and cultural norms as well as inherent disability (Kapp et al., 2013; Waltz, 2013). In taking this view of autism, perceived neurocognitive disadvantages become differences that may be advantageous in enabling contexts (Kapp, 2020). One social movement that has been particularly provocative in changing conceptualisations of autism and autistic people is the neurodiversity paradigm (Singer, 1998, as cited in Milton et al., 2020). This paradigm focusses on equal human rights for those with neurologically divergent conditions such as autism, and contests the idea of neuronormativity (Singer, 2016). Instead, the neurodiversity paradigm follows the view that all human brains and resulting perceptions differ to a degree (Milton, 2020). It is therefore proposed that each individual has a unique processing profile that cannot be grouped into a singular socio-cognitive framing (Milton, 2020; Mueller, 2020). As a result, those who would otherwise be framed as neurotypical are instead viewed as those who find dominant social constructs and norms to be enabling (Murray, 2020). Similarly, attention is drawn to the unique differences between autistic people that are often lost when summarising autism as a condition (Kapp, 2020; Milton, 2020). However, the paradigm also acknowledges the presence of a sense of shared culture and identity that has emerged for many within the autistic community (Kapp, 2020). Furthermore, with the neurodiversity movement has come an increase in autistic self-advocacy, encouraging a focus on the lived experiences of autistic people in framing what it means to be autistic (Bottema-Beutel et al., 2021). As a result, autistic people are increasingly involved in developing research enquiries and subsequent understandings of autism (Wright et al., 2014; Fletcher-Watson et al., 2019).

One theory in particular that has led to a positive-reframing of how we think about autism is Milton’s (2012) double empathy problem. The double empathy problem contests the view that autistic people have a theory of mind deficit, and instead draws attention to difficulties of reciprocity and mutuality between autistic and non-autistic people (Milton, 2012; Milton et al., 2018). Although these difficulties can occur between any two people, it is believed that the social realities of autistic and non-autistic people are more likely to differ, resulting in common two-way perspective taking difficulties (Milton, 2012). It is further argued that because a lack of social reciprocity is regarded to be relatively uncommon or easily repaired within non-autistic interactions, then autistic people must be to blame for breakdowns of reciprocity within an autistic - non-autistic interaction (Milton, 2012; Chown, 2014). Research on mixed-neurotype interactions have supported the double empathy problem, finding that non-autistic people recognise fewer autistic facial expressions (Brewer et al., 2016); struggle to identify autistic mental states (Edey et al., 2016; Sheppard et al., 2016); overestimate how helpful they are during communication with autistic participants (Heasman and Gillespie, 2019); and perceive a reduced sense of rapport compared to same-neurotype pairings (Crompton et al., 2020c). Furthermore, research has indicated that when autistic people interact with other autistic individuals, they may share some of the same-neurotype advantages observed within non-autistic pairings. Specifically, research has found that autistic people are more socially comfortable with other autistic individuals (Crompton et al., 2020a; Morrison et al., 2020); communicate information more efficiently (Crompton et al., 2020b); have a better understanding of each other’s social intentions (Heasman and Gillespie, 2018); and show an increased willingness to overcome initial negative impressions (DeBrabander et al., 2019). However, findings have indicated that autistic individuals may not have the same-neurotype advantages for perspective taking that are seen for non-autistic individuals (Brewer et al., 2016; Edey et al., 2016). While deficit-models would attribute this to an autism-specific theory of mind deficit (Baron-Cohen, 1997), it is possible that autistic people make more open-ended assessments of mental states that avoid premature conclusions. This is a reasonable suggestion since autistic people are more experienced in dealing with the lack of mutuality experienced within mixed-neurotype interactions that are commonplace for autistic people (Chown, 2014; Milton, 2020). Such a suggestion is consistent with autistic individuals taking more time to establish mutual social understandings while being less likely to draw rapid, heuristic-based social judgements based upon an assumption of pre-existing mutuality.

Research that explores the double empathy problem through a neurodiversity lens is important in challenging stereotypes toward the autistic community. Stereotyping, the holding of indiscriminate negative assumptions about individuals within a group (Kinnear et al., 2016), derives from the dominant model and deficit views of autism which reduce all autistic people and their experiences down to shared categorical impairments (Green et al., 2005; Pearson and Rose, 2021). This negative stereotyping leads to a polarising “us and them” assessment that further disadvantages autistic people (Goffman, 1990; Cage et al., 2018; Pearson and Rose, 2021). Importantly, this process called “othering” is a component of stigma that often results in discrimination and felt stigma (Goffman, 1990; Link and Phelan, 2001). The resulting felt stigma is reported by parents of autistic children (Gray, 2002; Mak and Kwok, 2010; Liao et al., 2019), as well as by autistic individuals themselves (Shtayermman, 2009; Griffith et al., 2012; Pickard et al., 2018). The stigma toward the autistic community is enhanced for those with intersecting identities, such as autistic individuals from racialised minorities (Broder-Fingert et al., 2020; Spense, 2020). These stereotyped and stigmatising views of autistic people further contribute to the socio-communicative breakdowns reported by the double empathy problem (Sasson et al., 2017; Pearson and Rose, 2021).

By contrast, methodologies that promote neurodiversity framings of autistic people are more likely to draw attention to individual differences, overcoming stereotyping and aiding double empathy (McCreadie and Milton, 2020). When assessing which methodologies to use for this purpose Ida’s (2020) theoretical assessments around multiplicity and neurodiversity should be considered. Specifically, Ida (2020) argues that methodologies which afford openness to multiple possibilities should be favoured. Where this multiplicity is achieved, individuals look beyond their separate identities to assess how their differences are constructed (Ida, 2020; McCreadie and Milton, 2020). These assessments of individual differences are believed to be facilitated by shared experiences that enable a dismissal of coarse group-based understandings (Ida, 2020). Additionally, explorations of the nuanced difference within wider similarity are important to overcome the double empathy problem (McCreadie and Milton, 2020; Mueller, 2020). Furthermore, it is argued that strictly scientific research methodologies should be avoided to prevent reliance on binary, neuronormative ideologies (Ida, 2020; Mueller, 2020). Instead, creative and open methodologies that provide an immersive shared experience are more likely to afford multiplicitous, double empathy understandings (Mueller, 2020).

One potential methodology that would afford this type of multiplicitous thinking is the discussion of fiction. This is because the shared reading of fiction promotes communal thinking about a text, whilst also enabling explorations of individual differences within (Longden et al., 2015). Additionally, it is argued that fiction is inherently social, drawing on three levels of perspective-taking or “theory of mind”; (1) the mind of characters, (2) through the mind of the author, and (3) through the mind of the reader (Zunshine, 2011). In this way, shared discussions around fiction may add a 4th level of perspective-taking, exploring the first three levels through the interaction with other readers and thus other minds (Longden et al., 2015). While the first three levels provide a shared experience that results in communal thinking, it is the fourth level that is important for the shared exploration of individual differences. Additionally, it is believed that in the act of reading readers infer emotions and perspectives through the evocation of past, personal memories that promote more mindful self-other comparisons (Mar and Oatley, 2008; Mumper and Gerrig, 2019). This means that shared reading may be a particularly advantageous methodology for autistic people because it engages the ability to make more open-ended and in-depth assessments of perspective. Importantly, the social simulations of fiction are believed to inform real world understandings (Mar and Oatley, 2008; Mumper and Gerrig, 2019). Therefore, any understandings that are developed toward autistic individuals through the contemplation of fiction should result in broader understandings of the autistic community. As a result, shared fictional reading becomes a potentially useful tool in overcoming the double empathy problem.

It is argued that serious literary fiction is the most provocative form of fiction for eliciting empathic understandings of different perspectives, where serious literature refers to literature that engages with significant human situations and as a result enables its readers to do the same (Koopman and Hakemulder, 2015; Davis and Magee, 2020). It is the powerfully moving language of serious literature which is important in this regard because it jolts people out of normative, stereotyped thinking patterns (O’Sullivan et al., 2015; Davis, 2020). Furthermore, serious literature requires the consideration of multifaceted, often ambiguous, meanings within complex social constructs that are not conducive to the drawing of hasty conclusions (Mar and Oatley, 2008; O’Sullivan et al., 2015; Davis, 2020). Reading aloud methodologies incorporate this shared contemplation of serious literature (Longden et al., 2015). Within these groups, the liveness that results from reading aloud results in strong absorption and felt unpredictability that promotes complex literary assessments (Longden et al., 2015; Davis and Magee, 2020). While this type of methodology may be advantageous in overcoming the double empathy problem, research has highlighted that some autistic people are uncomfortable with the idea of reading in a group and being read aloud to (Chapple et al., 2021). Instead, the value of shared reading within pairs of autistic and non-autistic individuals may be more tolerable as well as more likely to elicit double empathy understandings.

The current study qualitatively explores changes in understanding and the double empathy problem between autistic and non-autistic participants as a result of shared reading discussions. Specifically, participants read and subsequently discussed John Steinbeck’s novella, *Of Mice and Men* (1937). This book offers a provocative shared experience, with multiple examples of stigma toward minority groups, bringing the necessary consideration of difference to the forefront (Ida, 2020). To account for the concerns of autistic people in participating in groups, the study focussed on pairs of autistic and non-autistic individuals. Furthermore, in place of live readings, participants completed a structured diary entry per chapter which were subsequently used as discussion aids. The study aimed to address the research question: “can discussions of literary texts involving autistic and non-autistic people overcome the double empathy problem and result in empathic understandings of one another’s perspectives?”

## Materials and Methods

### Participants

Participants were recruited through social media and local advertisements into a wider project that included this study and an earlier, unpublished study upon which this one was built (see section “Procedure”). Initially, 20 participants, of whom 15 were non-autistic, indicated a willingness to be involved in the wider project. Due to the lower number of autistic volunteers, these participants were prioritised for study inclusion. Non-autistic participants were paired with autistic participants based on gender and, where possible, age and educational background. Five pairs had been intended for inclusion. However, one autistic participant dropped out of the study due to time restrictions, resulting in four pairs. The decision was made not to include a fifth pair due to having achieved data saturation; a result of the longitudinal nature of the research, with each participant contributing 15 to 16 pieces of qualitative data. Inclusion criteria included being 18 or over, having proficient English language skills, and being able to travel to the University of Liverpool. Non-autistic participants had additional inclusion criteria of scoring below 32 (the suggested cut-off score for autism) on the autism quotient (AQ; Baron-Cohen et al., 2001) due to potential trait overlap. Two non-autistic participants who identified as dyslexic were permitted inclusion into the study. This was because the participants identified as neurotypical rather than neurodivergent, and were comfortable with the reading, writing, and comprehension that the study required. Autistic participants had no additional exclusion criteria, as all participants reported a formal diagnosis and none reported learning difficulties that might have resulted in altered comprehension or difficulties in reading and discussing the text.

Overall, 8 participants (see Tables 1, 2 for demographics), within 4 participant pairs, took part in this study. The 4 autistic participants comprised 2 male and 2 female participants aged 19–48 (*M* = 30.25, SD = 12.53). The 4 non-autistic participants also consisted of 2 male and 2 female participants that were aged 23–33 (*M* = 28.75, SD = 5.06). It happened that all pairs comprised 1 participant from a racialised minority and 1 who was of white British nationality. Data on race and nationality was not formally collected from participants but was raised by participants themselves within the qualitative discussion sessions. Of those who were from a racialised minority, 3 were autistic and 1 non-autistic. All 8 participants were invited to a follow-up interview with 1 non-autistic participant not providing a follow-up interview. The study was approved by the University of Liverpool Research Ethics Committee.

**TABLE 1:** Participant AQ and IQ scores between neurotypes [mean(± SD)].

	AQ^*a*^	Estimated IQ^*b*^ (WAIS equivalent)
Autistic	40.35 (6.24)	98.50 (6.81)
Non-autistic	11.75 (1.26)	102.50 (3.79)

*AQ, autism quotient; QT, quick test; WAIS, wechsler abbreviated scale of intelligence. ^a^AQ scores. ^b^IQ assessed by the QT.*

**TABLE 2:** Participant demographics.

Pair no.	Participant no.	Age	Gender	AQ^*a*^	IQ^*b*^ (WAIS equivalent)	Level of education completed	Neurodiversity status
1	1	29	Male	42	96	GCSE	Autism diagnosis
1	7	23	Male	10	100	Masters	Identifies as neurotypical
2	8	26	Female	12	102	Bachelors	Identifies as neurotypical
2	20	19	Female	31	92	A level	Autism diagnosis
3	9	33	Female	12	100	Doctoral training	Neurotypical
3	11	48	Female	44	108	Doctoral training	Autism diagnosis
4	10	33	Male	13	108	Foundation or diploma	Neurotypical
4	18	25	Male	44	98	Masters	Autism diagnosis

*AQ, autism quotient; QT, quick test; WAIS, wechsler abbreviated scale of intelligence. ^a^AQ scores. ^b^IQ assessed by the QT.*

### Screening Measures

A demographics questionnaire asked for participants’ age, gender, and highest completed qualification. Eligibility questions were also asked at this stage.

#### The Autism Quotient (AQ) (Baron-Cohen et al., 2001)

The AQ is a 50-item questionnaire that uses statements to elicit a score that reflects autistic traits in clinical and non-clinical samples. The AQ was used to assess the number of self-reported autistic traits in both samples.

#### The Quick Test (QT) (Ammons and Ammons, 1962)

A single 50-item version of the QT was used to quickly assess the comprehension abilities of participants, a factor that was considered important within a methodology that relies on text comprehension.

### Session and Interview Measures

#### Participant Diaries

As part of the preceding study (see section “Procedure” for further details), participants read *Of Mice and Men* (Steinbeck, 1937) at a rate of 1 chapter per day for 6 days. For this study, diaries were returned to participants as optional conversational prompts.

For each chapter participants were asked to answer the same 5 questions. Questions 1 to 3 were designed to prompt general reflections about narrative events and characters: (1) *what thoughts or feelings did chapter X prompt?* (2) *do you think the characters in chapter X were realistic?* (3) *did you like or dislike the characters in chapter X?* Questions 4 and 5 were added based on previous findings that autistic readers think more about author intent (Chapple et al., 2021): (4) *did you think about the author when reading chapter X?* (5) *what did you think the author was trying to achieve in chapter X?* In the current study, these 5 questions served as optional conversational prompts during the discussion sessions (see section “Procedure” for further details on the sessions).

#### Pre-session Questionnaire

A pre-session questionnaire was designed to explore participant views on the group which they did not identify with (neurotypical or autistic). Participants were asked (1) to define what it meant to be autistic/neurotypical as appropriate, (2) how they think the two groups differ, and (3) why they chose to take part. To take account of familiarity with autism, the non-autistic group were asked whether they personally know an autistic person.

#### Post-session Questionnaire

A post-session questionnaire was designed to evaluate participant thoughts after each session. Participants were asked (1) what things (if any) were discussed about the book or diaries, (2) what things (if any) were discussed outside of the book or diaries, (3) whether the discussion helped them to understand the other participant better, (4) whether they gained any self-understanding, (5) whether they enjoyed the session, and (6) whether their understanding of autistic and neurotypical differences and social interactions had changed as a result of being involved in the discussion sessions.

#### Interview Schedule

For the 7 participants who chose to take part in the follow-up semi-structured interview, this occurred at least 1 week after their final shared reading session. During the interview, participants were asked about (1) whether they had benefitted from being involved in any way, (2) what they thought of the sessions, (3) if and how their understanding changed toward the other group, (4) whether the study helped their self-understanding, (5) if they felt the other member of their pair had sensitively understood them and the group they identified with, (6) how they would now define the other group, and (7) if anything could have been added to the study that they felt could have improved personal outcomes. The schedule was made up of structured, open questions and follow up questions.

Dictaphones recorded the interviews which were subsequently manually transcribed by the first author. All field notes and questionnaires were also converted into Word documents. Documents were uploaded to NVivo 10 (Castleberry, 2014) to facilitate analysis.

### Procedure

Potential participants completed a screening process using the Qualtrics online platform. It included the informed consent procedure, a demographic questionnaire, the QT and the AQ. Participants who screened out based on the exclusion criteria, or who did not leave an email address for contact had their data destroyed. Non-autistic participants who screened in were matched to the four autistic participants and invited into the study.

All 8 participants first took part in the connected study, in which they read *Of Mice and Men* (Steinbeck, 1937) while recording their thoughts in a structured diary. For this preceding study, participants read alone and did not meet with the partners that they were paired with for the current study. The diary was completed for 7 days, the first 6 coincided with reading one chapter of the book per day. On day 7, participants completed 3 writing tasks that prompted reflective thinking about the overall novel. For this preceding study, the participant diaries were analysed to assess whether autistic and non-autistic participants engage with serious literature in similar ways. in the current study, the book and diaries were instead used as conversational prompts for the shared reading sessions.

The discussion sessions occurred weekly for 4 weeks and lasted for 1 h. Two of the participant pairs attended the four sessions in-person in a designated, quiet interview room at the University of Liverpool. The other two pairs took part via Skype due to COVID-19 imposed restrictions at the University. Before the first session, participants completed the pre-session questionnaire. During the informed consent procedure, it was explained to participants that the lead researcher would be present for the full duration of the session and could offer assistance of any kind. However, the researcher otherwise remained silent during these sessions, and participants were made aware that the researcher would not be involved in the discussions. For the in-person sessions, the researcher sat at the other end of the room, in peripheral view of the participants. For the Skype sessions, the researcher remained visible via webcam to try to replicate the in-person discussion sessions. The physical presence of a researcher was incorporated into the study design to ensure discussions remained respectful and to enable note taking. In both settings, it was explained to participants that the researcher would take notes on discussion topics. Field notes were recorded to summarise the topics being discussed within pairs. Where participants were having back and forth discussions that were neither summarising *Of Mice and Men* (Steinbeck, 1937), or repeating their diary responses, the researcher made direct transcriptions of the dialogue between participants. Field notes and direct transcriptions were chosen to record the session content as opposed to audio or video recordings because it was felt to be less intrusive. Participants were given their individual reading diary at the start of each session and instructed that they could discuss anything, whether related to the book or not and so were allowed to structure their own sessions. Participants were reimbursed £10 for involvement in each study component.

The first author is an autistic, female Ph.D. researcher, who is trained to Master’s level on semi-structured interviewing. The first author facilitated all of the discussion sessions and conducted all 7 of the follow-up interviews, with no other researchers present. All autistic participants were informed that the facilitating and interviewing researcher would also be an autistic adult. The researcher was acquainted with two of the autistic interviewees but was unfamiliar with the other six participants.

Participants were later sent the results from the study and invited to provide feedback. Participants were specifically asked (1) “*do you have any thoughts about how we’ve understood your data?”* (2) “*Have you thought about the sessions since the study?”* (3) “*What things about the study have felt important since?”* (4) “*Has your experience of being involved in the project altered how you approach daily communication?”*

### Analysis

SPSS and Microsoft Excel were used to organise and calculate descriptive statistics and scores from the screening questionnaires.

Interviews were transcribed using edited transcription, with the omission of irrelevant false starts, filler sections and repetition, unless used to convey importance or significance. Transcription was completed by the first author who has prior experience of interview transcription for post-graduate research. Resultant transcripts were not sent back to participants as there were no areas of unclarity or missing data due to poor sound quality. One participant was sent their pre-session questionnaire and first post-session questionnaire due to unclear data, this process resulted in recovery of the main points within the data. Qualitative data from session questionnaires, researcher field notes and interview transcripts were analysed primarily using thematic analysis (Clarke and Braun, 2014), with a combination of Framework Analysis (Ritchie and Spencer, 1994) and a form of literary close reading analysis (Billington et al., 2019). The first two stages of Framework Analysis (immersion and organisation) were implemented using NVivo 10 (Castleberry, 2014) due to the rigour of these particular stages that reduced data loss, making it ideal for the longitudinal nature of the data. After this stage, rather than implementing the re-coding process that follows in Framework Analysis, the team switched to a manual thematic analysis to group data into themes. This shift, implemented in stages three and four, was chosen because thematic analysis better enabled the articulation of the narrative flow of the data itself and the inter-disciplinarity of the research. Finally, a form of literary close reading analysis (Billington et al., 2019) was implemented in stage five that relies on participant language as “the main point of access to moments of subtle mental change” that give access to the “imprints” of reading (Kaszynska, 2015). These qualitative analyses combined to ensure a deep and rich exploration of the data, necessary to explore the complexity of human interaction mixed with literary explorations across time. As a result, analysis stages were as follows:

(1) The first author transcribed the raw questionnaire and field note data, and the 7 interview transcripts, followed by a first reading of all data with memo creation for data immersion. The second, third and sixth authors reviewed data from one pair for immersion.
(2) The first author sorted all data into an initial, organisational framework within NVivo 10. Initial ideas were discussed with the rest of the team and the organisational framework was reorganised accordingly.
(3) The first, second, third and sixth authors deliberated on the organised categories and identified four themes. Themes were refined through continued discussion and exploration of the data examples within each theme.
(4) The researchers picked out key quote examples from the data for each theme and sent these quotes grouped into the four categories without labels to the fourth and fifth authors for review. Upon agreement of the categories, the authors were then sent theme names and explanations for review.
(5) To further explore each thematic outcome, the second and third authors, experienced in the literary analysis of texts and participant responses, applied a literary close reading analysis to the data examples chosen by the team for each theme. This final analysis was then reviewed by the rest of the research team for approval.

The first author is an autistic researcher. Additionally, the fourth and fifth authors are autistic adults who were invited to join the research team as experts by experience. These authors were consulted on the analysis as detailed above, as well on the theoretical framings and language used within the paper. Where the fourth and fifth authors raised concerns with regards to the analysis or wider paper framings, alternative framings were agreed. As a result, all data was analysed and subsequently understood from autistic and non-autistic perspectives.

## Results

### Pre-session Questionnaire Summary

Of the non-autistic participants, two reported no personal link to an autistic person, one reported a professional link, working with autistic children but not adults, and another reported that their partner’s relative is autistic.

The most common reason overall for engagement with the study was interest. Half of the non-autistic participants additionally reported getting to hear the lived experience of an autistic adult as a motive. In comparison, half of the autistic participants reported the ethos of the study in meeting wider autistic community goals as a motive. Additionally, financial reimbursement and self-exploration were listed as unique, individual motives.

### Qualitative Analysis Results

The final analysis comprised four themes: (1) the book as social oil (2) from a world of difference to a world of affinity (3) emotional intelligence: from thinking about to feeling with, and (4) from overwhelming to overcoming. Participant quotes are split by neurotype group (A: autistic, N: non-autistic), and by timeframe (S0: pre-session; S1–S4: discussion sessions in order; S5: final interview).

#### The Book as Social Oil

Although participants were free to discuss any topic of their choosing during the sessions, all pairs centred their discussions on the book and their associated diary responses. In this way, the text acted as a meaningful shared experience for participants to begin their dialogues. That both readers knew the book and its characters, was reported by participants as having reduced the usual social awkwardness often felt on first meeting:

*(P11A) [S5] “actually having a topic that you could talk about and around helped. I think if we’d have just gone in a room and said “right, chat” then there would have been a lot of awkward silences”*

*(P8N) [S5] “it’s less awkward ‘cos you’ve got like prompts [the literature] gives you a conversation starter, save any like awkward silences.”*

Although this initial reduction of social awkwardness stemmed from the book serving as common ground, the narrative additionally provided a shared social setting to operate within during discussion sessions: hence discussion was not just “about” but “around” and “within” the book. Through participant discussions, characters were further *brought to life* as complex, social beings in a developing relationship. The involvement of the readers within this shared immersive experience created more in-depth personal and social discussions, with the perceived safety of the simulated social setting affording more risk-taking:

*(P7N) [S5] “I think it was a good introduction because it allowed you to go into other topics, ‘cos kind of just asking somebody off the bat “how would you feel in this situation?”*… *people would be a bit more defensive. But I think it was a good introduction of “how would you act in the situation of that character?” And then a conversation expanded from that into the more mundane aspects of your life”*

*(Pair 4) [S1] P18A: “I dislike George condescending [to] Lennie*…, *however, it does frustrate me that Lennie doesn’t know his own strength. I like and dislike them both in different ways.”*

*P10N: “I’d agree with this, Lennie has good intentions but it results in bad consequences”*

Where social difficulties arose, both participants within the dyads showed an ability to sensitively overcome these difficulties by bringing the focus back to the novel to move discussions on. Difficulties included times when discussions became circular in nature, where long periods of unintentional silence occurred, and where participants expressed uncertainty about how to move discussions forward. Primarily and at least initially, non-autistic participants had wider concerns about dominating conversations, while autistic participants desired more social guidance. This resulted in participants instinctively implementing a planned structure, drawing on the structure of book chapters and diary questions to alleviate their mutual concerns and difficulties:

*(P18A) [S1] “the other participant gave me cues to speak and to guide me on which parts we should talk about next. I felt this was especially helpful as it maximised my potential in being able to contribute to the conversation as effectively as possible”*

*(P10N) [S5] “we almost set out a plan. We knew we had four sessions, “we’ve got this many chapters, these many activities, we’re going to kind of split it up like that.”*…*so, we kind of knew from the off what the plan was*…*what I personally didn’t want to do was lead every single question, and then he feels like he had to kind of give an answer that was similar to mine. So, we took it in turns”*

As a result of the shared social setting afforded by the book and the creative overcoming that resulted from times of social difficulty, autistic participants reported feeling valued within discussions. Importantly, they reported that even when their views differed from their partner’s, they felt their views were considered and valued, rather than socially ill-fitting:

*(P20A) [S4] “[session discussions] made me realise that my interpretations of themes throughout the book are just as valid as other interpretations, and therefore my perspective is not necessarily wrong.”*

*(P18A) [S5] “what I found more interesting, was he found them to be acceptable, he found my reasons to be valid, just as much as I thought that his reasons were also valid.”*

Contemplation of the book and diary reflections resulted in an openness within pairs. This openness enabled the pairs to explore their nuanced *differences* of reasoning within the context of their shared experience, wider similarities and shared conclusions as readers. In this way, the literature brought their attention to their more subtly and freely found understandings of the text. This moved participants away from thinking about their categorical neurotype differences, toward a focus on their individuality within the experience of shared reading:

*(P7N) [S5] ‘we had mutual agreement on a lot of things and what we reflected on was quite similar*… *an ice breaker to go “you know what, we’re not actually that different because we haven’t looked at this and gone miles apart. Our reflection on this piece of literature was similar.”’*

*(P20A) [S5] ‘I realised “oh, there are some similarities between us because we’ve written different things but in similar ways.”’*

#### From a World of Difference to a World of Affinity

With the shared experience and perspectives thus afforded by the literature giving participants a unifying structure within which to explore their differences, the sessions provided room for participants to explore the bidirectional nature of their differing world views:

*(Pair 1) [S2] P7N: “Why were you so focussed on the dog being shot [in the narrative] as an upsetting event?”*

*P1A: “I do have a liking for dogs, and I wish he’d just simply given the puppies away.”*

*P7N: “I can understand them being shot, in these circumstances, the dogs would have died painfully.”*

*[Researcher: P1A doesn’t reply but appears to be at ease about the narrative events after this]*

*P7N: “Have you ever had rabbits?”*

*P1A: “No, I’ve only ever had a hamster.”*

*P7N: “I’ve had rabbits, they bred a lot and so I had to drown them. I also used to shoot rabbits, hunting them was a hobby. We’d eat them afterward, they were tasty, but we had to stop hunting because a local illness wiped the rabbits out.”*

*[Researcher: P1A doesn’t reply but looks visibly uncomfortable]*

Where wider differences and associated social discomfort had arisen, participants had to work harder to find common ground outside of the shared narrative experience. Participants identified these additional common grounds by re-visiting their shared opinions within the novel, and looking to real-world situations where these opinions translated into a contemporary situation. For example, participants 1A and 7N assimilated their dislike of the aggressive behaviour observed from the character *Curley* to that which they mutually disliked seeing displayed by others in their local areas. Their experiences of such aggressive behaviour being directed onto them in real life then served as new common ground to return to when wider differences of opinion presented. These explorations of common ground still served to move participants away from focussing on the anticipated differences based on neurotype. Therefore, participants were further moved toward understanding each other as sharing these specific human experiences. For the non-autistic individuals, a reframing of their understandings of autistic people emerged that moved away from a focus on basic difference, toward a focus on the emergent recognition of essential similarity:

*(P7N) [S5] “it’s not a case of “us and them” it’s more of a “hang on we agree on a lot of things we’re just slightly different.” As opposed to “they’re miles apart” I think that’s probably changed.”*

This focus on essential partner similarities within pairs provided the scaffold to enable the deeper exploration of the nuanced differences that existed between them: “slightly” rather than “miles apart.” All dyads reported that the differences that existed between themselves and their partner were actually subtle and contextual:

*(P11A) [S5] “I think as people we probably had a fair amount in common*…*I think our backgrounds are quite different, so she’s obviously a lot younger, a lot more widely travelled, she seems to have lived a very straight forward life.”*

Here, “more widely travelled” but “very straight forward” seems itself to be a subtle account of a particular form of ease that P11A lacked.

*(P10N) [S5] “what it probably showed me was that there’s probably a lot more similarities than differences, and the differences tend to be a little more subtle than I probably would have expected them to be.”*

Through (1) establishment of common ground, followed by (2) explorations of the finer differences, participants (3) moved away from constricting over-simple assumptions based on neurotype. Instead, participants started to view each other as suitably complex individuals:

*(Pair 4) [S4] P10N: “Our focus on society in the sessions has showed that we have more similarities than differences. It felt no different to socialising with my friends, and if I’d not known you were autistic, I’d have just thought we were different people individually”*…

*P18A: “I don’t feel we are different from each other by much now, despite our neurological differences”*

*(P11A) [S5] “I was surprised how similar our perspectives were*…*I didn’t really see it as a neurotypical and an autistic way of thinking.”*

What P11A articulates above is a sense of surprise, relief and pleasure in the fellowship that emerged.

#### Emotional Intelligence: From Thinking About to Feeling With

A key factor in non-autistic participants developing a more sensitive understanding of their autistic partners was the lived experience accounts that remained at the forefront of discussions throughout the study. Rather than starting from a deficit view and seeking to identify difference, these accounts, which were often proffered in the context of humane discussion of the literary events, enabled non-autistic participants to learn from their partner’s explanations and experiences of what it means to be autistic:

*(P7N) [S5] ‘The lived experience is different from the dictionary definition. So, I kind of feel if we went into it with a dictionary definition, we may just start to categorise people from the offset “well he said that, that roughly correlates to this, so oh yeah that’s definitely autistic.” I suppose going into it from a bit more of a personal opinion kind of thing, to be quite frank more of a position of ignorance, helped to inform me better, ‘cos I think if I went in knowing loads of stuff about autism on paper I would have just went “yeah, his reaction to this means he’s got this trait.”’*

*(P10N) [S5] “anyone can read a definition of something and kind of spout it out. But I think the best thing if you want to actually understand somebody is to actually go and find out for yourself really, and actually speak to somebody”*

The literature is what took these participants beyond literal, dictionary definitions into a more imaginative and emotional pooling of experience. While the lived-experience nature of the sessions encouraged the development of emotional intelligence toward autistic participants, it was the literature which brought autistic and non-autistic participants to feel with one another. The emotionally provocative events within the narrative encouraged participants to share their own emotional experiences of reading the text:

*(P20A) [S4] “I cried a lot, the shortness had a bigger impact, due to there being so much to process in so little time then having to move on.”*

*(Pair 3) [S3] P9N: “I felt too sad during this chapter, with the bad events for the characters.”*

*P11A: “It was sad, it felt like a slow-motion car crash, you knew what was coming so everything felt slower”*

Through these shared exchanges, participants began to process their own each other’s emotional reactions to the text, exploring the depth behind their emergent feelings. Specifically, the discussions brought their earlier emotional reactions forward into the session in reactivated memory, allowing them to feel through the experience again. This resulted in explorations of what contextual factors had elicited their complex reactions. Through this individual processing of text reactions within discussions, participants were then able to comparatively explore their different understandings, feeling through their emotions together. This was often through exchanges of one participant offering complex insight that evoked surprised silence from their partner, as they processed the depth of the emotions brought forward through the narrative:

*(Pair 4) [S3] P11A: “I found it peacefully surreal [the death of Curley’s wife and looming death of Lennie], during distress there are brief moments where you forget and have moments of peacefulness.”*

*[Researcher: P9N seems surprised by this.]*

Stigma in particular was a recurring point of discussion between pairs, reflecting the experiences of narrative characters. The book acted as a key social catalyst in this way, with complex examples of stigma toward multiple minority groups, resulting in in-group stigma amongst marginalised characters. In particular, participants tended to feel empathy *with* the character Lennie, together. Lennie is a character who was discriminated against by other book characters for his unnamed neurocognitive disability. These empathic responses also resulted in shared frustrations toward characters who mistreated Lennie:

*(P18A) [S2]: “the dream [of character’s getting their own farm] feels more real now and it makes me worry for Lennie because I empathise with how he’s bullied and how Lennie wants to avoid trouble but George is giving him opposing advice.”*

*(Pair 3) [S2] P11A: “I couldn’t understand Curley and why he’d hit Lennie if he [Lennie] wouldn’t hit back”*…*[S3] P9N: [talking about why Lennie responded to the death of Curley’s wife the same as he did a mouse] “I think Lennie was scared of George, he relies on him and didn’t want to disrupt harmony.”*

This evocation of empathising with Lennie resulted in the dyads engaging in further complex, emotional discussions of the text. For P20A and P8N this resulted in questioning the surface assumption that Lennie needs George to survive, by imaginatively and sensitively going further to consider the mutuality of this dependence:

*(Pair 2) [S1] P20A: “I wonder if George would survive without Lennie and if Lennie would be better off without George?”*

*P8N: “I think Lennie would find someone else*…*”*

*[S3] P20A: “George doesn’t help himself by hiding it” [Lennie’s disability]*…

*P8N: “I don’t think George wanted him to be seen or treated as different, but maybe that’s why he keeps getting in trouble.”*

*P20A: “I think it shows how much Lennie and George need each other.”*

Here, the use of “I wonder” and “I think” shows signs of individual, imaginative risk-taking from P20A.

Similarly, all pairs expressed a feeling of mutually shared empathy *with* the character Crooks, who experienced both racial and physical-disability related discrimination. In comparison to Lennie, these feelings were more conflicted, holding in mind a frustration with how Crooks stigmatised Lennie for his disability and at the same time feeling through the difficult emotions that resulted in Crooks behaving this way. This tended to lead to further evaluation of what role Crooks served as a literary device. Below is a short passage showing the interaction between Crooks and Lennie, followed by participant responses:

*(A passage from Of Mice and Men of Crooks and Lennie meeting; Steinbeck, 1937)*

Noiselessly Lennie appeared in the open doorway and stood there looking in, his big shoulders nearly filling the opening. For a moment Crooks did not see him, but on raising his eyes he stiffened and a scowl came on his face. His hand came out from under his shirt.

Lennie smiled helplessly in an attempt to make friends.

Crooks said sharply, “You got no right to come in my room. This here’s my room. Nobody got any right in here but me.”

Lennie gulped and his smile grew more fawning. “I ain’t doing nothing,” he said. “Just come to look at my puppy. And I seen your light,” he explained.

“Well, I got a right to have a light. You go on get outa my room. I ain’t wanted in the bunkhouse, and you ain’t wanted in my room.”

“Why ain’t you wanted?” Lennie asked.

“Cause I’m black. They play cards in there, but I can’t play because I’m black. They say I stink. Well, I tell you, you all of you stink to me.”

*(Pair 2) [S3] P8N: “I found Crooks the most interesting, it’s interesting that he gets his own chapter.”*

*P20A: “Why did he?”*

*P8N: “There’s a lot about race, and that sometimes is sympathetic but also Crooks can be horrible. You start disliking Crooks, then feel sorry for him because he’s got the worst life.”*

*P20A: “It shows there is depth to these people, which is why the author took time to speak about him”*

*(Pair 3) [S2] P9N: “I felt sad for Crooks due to the racism he endures*… *he’s denied simple pleasures such as living with others or being involved in games. I think the racism was deep rooted, with him seeing Lennie as intruding and being fearful of others and losing his job, despite the fact that Lennie was too naïve to consider this. I think Crooks is safety-focussed.”*…

*P11A: “Crooks would have known the risks and likelihood of being blamed, resulting in avoidance and constant terror. He could have had a nice friendship with Lennie, as Lennie would have had no prejudice against Crooks.”*

By bringing the realities of complex emotions forward into discussions, the literature encouraged participants to process their own lived experiences of similar events, such as stigma and grief. These experiences were shared within pairs, drawing parallels to narrative events. While participants had already began to mutually feel with one another, these discussions of stigma tended to be unfamiliar for non-autistic participants. However, with the prior evocation of empathic responses elicited by similar events within the literature, non-autistic participants were moved from feeling for to feeling with their partners, although unfamiliar experiences were being disclosed. Conversely, where both participants had a shared, personal experience, disclosure from one resulted in empathic disclosure from the other:

*(Pair 1) [S4; after discussing the racism toward Crooks in the book] P1A: “When I was in a choir, as a child, I experienced racism”*…

*P7N [shocked]: “Who would be racist to a child?”*

*P1A: “Multiple teachers disliked me and I’m unsure now if it was due to being autistic or if they were being racist.”*

*(Pair 3) [S1; after discussing their empathy toward Candy for having his dog put down in the book] P11A: “I had to put my dog down and that results in complex emotions”*

*P9N: “I had to put my cat down, it is difficult when you know your pet is suffering.”*

#### From Overwhelming to Overcoming

Individuals generally had to overcome over-simple or stereotype-based concerns or barriers that presented between themselves and their partner. For autistic participants, their concerns toward non-autistic people in general were centred upon past experiences of being stereotyped and stigmatised. These concerns were factors that contributed to social concerns *before* participants had met with their non-autistic partners:

*(P1A) [S5] “they have a stereotype in their mind, whether it’s due to you know the odd film or what they’ve seen briefly in real life and they don’t fully grasp and understand. They think a lot of the traits are tied to all autistic people whereas obviously it varies”*

In contrast, the non-autistic group had to overcome previously held general concerns of difference in relation to autistic people:

*(P10N) [S5] “maybe I overestimated the impacts that it [being autistic] would have on what I would deem to be like a normal life*… *At the end of the day, whether you’re diagnosed with something it’s kind of, it doesn’t really matter, everyone’s different, everyone’s going to take different things from it*… *you’re going to have to take everyone on their individual face-to-face I suppose. So, I suppose it’s not being quick to kind of type-cast somebody”*

Part of this difficulty was that non-autistic people were viewed generally by autistic participants as not having to face and overcome social difficulties in their day-to-day lives because they belong to the majority neurotype. However, the literature dismantled this over-simple generalisation within pairs by introducing social overcoming. As a result, *both* autistic *and* non-autistic participants showed evidence of having to overcome social challenges drawing on the felt affinities between the literary characters and themselves to do so:

*(P8N) [S5] “I thought it was interesting when the participant [20] was saying that they felt more of an affiliation with Lennie, ‘cos I guess if I was thinking about it, I probably would feel more of an affinity with George overall.*

*[SI] George’s stubborn and resentful attitude makes him harder to like.”*

*(Pair 4) [S3] P18A: “I don’t know why George done that to his so-called friend, but I feel he regretted it*…*”*

*P10N: “I felt George had no choice*…*”*

*P18A: “I might have done the same if I was George”*

The complex reflective statement from P8N indicates that the affinity with George was not one of liking and, in the vein of overcoming, its relation to the participant’s own rather critical self-judgement was clear. Similarly, for P18A the shifts and modifications and overall mobility are evidence again of a more complex to-and-fro interaction.

During the first couple of sessions, social difficulties sometimes occurred as participants worked to overcome their differences. While these difficulties tended to centre on minor social discomfort and general awkwardness around continuing to-and-fro conversations, for participants 1A and 7N, there were incidents in the second session of conflicting emotional opinions. This conflict felt overwhelming for P1A, as we have seen. These events stemmed from P1A sharing feelings of unease toward the event in the book which he later felt was not responded to empathically by his partner:

*(P1A) [S5 – recalling events from S2] “I kept referring to my distaste for a certain character for drowning puppies, he in real life brought up in an almost gleeful manner that he’d drowned rabbits*…*that was kind of disturbing.”*

These isolated incidents of social discomfort between participants seemed to mirror the idea that non-autistics were not experienced in adjusting communication to take account of others. By contrast, autistic participants reported having regularly to adjust their communication in day-to-day life so as to overcome social difficulties that present during communication with non-autistic people. As a result of a so-called “deficiency,” autistic participants have to develop an advanced capacity to consider and hold in mind complex, alternative ways of being and perspectives:

*(P1A) [S0] “a lot of traits they [neurotypicals] have I either don’t relate to or can’t stand. Examples, small talk, can be two-faced. Whereas I envy not being able to cope better with sensory issues so there are positives too*…*though a favourite has to be bluntness which neurotypicals can lack.”*

It was a perceived lack of honesty, disguised through social skills, which P1A struggled with. The result as here is often a more complex mental syntax in response (“whereas. Though”).

For non-autistic participants, social overcoming exemplified within the text seemed to result in a wider acceptance of differing perspectives in participants working together patiently in real time outside it:

*(P10N) [S5] “it kind of made me re-evaluate that people can pick up different things and neither one is wrong*…*it’s just made me think about if something seems odd to me*…*then by taking a little bit of time to kind of chat to somebody and just kind of figure out their process, actually it makes it easier for me to understand how they’ve got to that point. I mean that works for autistic or non-autistic”*

As the discussion of lived experience contributed to the move from feeling overwhelmed by difference to the emergence of a will to overcome difference, supported by acknowledged similarities, so, taking time over the four sessions resulted in built rapport:

*(P7N) [S5] “I personally feel having that same person you got to build that relationship and you got to understand what our differences are better. I know it wouldn’t be a representative sample*…*but it allowed you to build a relationship in which you felt comfortable to talk about certain things. And I think by the time we got to session three, when we were on some of the shall we say more divisive aspects of the book; the racism, the murder, the sexism and discrimination with disability, you wouldn’t be able to necessarily discuss that with somebody you’d just met.”*

What emerged was genuinely “built” social connection within pairs and a positive desire to work on a social bond rather than concentrating on neurotype identities:

*(P10N) [S5] “I looked forward to seeing the participant, and kind of seeing what his take was*…*it almost got to the point where I didn’t think it was an autism study”*

This quote from P10N is testimony to the depth of connection achieved.

### Participant Feedback

Participants 10N, 1A, 11A, and 20A decided to provide feedback on the overall findings from the study one year later. Participant 1A reported reflecting on the study to consider how his partner viewed him as an autistic adult and how this might translate to the way non-autistic people view autistic people in wider society. However, participant 1A did not find any improvements in communication with non-autistic individuals outside of the research. Participant 11A reported continued reflections on the shared reading sessions and a resultant improvement in making her own intentions clearer for mutual understandings:

*(P11A) “Now, I try to think about how other people might view me and what I put across. I also try to explain my thinking/feeling a little more, although this can be difficult at times.”*

Similarly, participant 20A reported that the feeling of being valued in having a different perspective translated into her everyday life, making her feel more open herself toward differing perspectives:

*(P20A) “I have realised that my own interpretations of things are not necessarily wrong and there are different perspectives that you can respect. I have tried to be more open listening to what others have to say even if I do not agree.”*

Participant 10N reported the biggest changes in his everyday life as a result of taking part in the research. Importantly, the participant reported slower, more careful thinking in assessing the perspectives of others. As a result, the participant felt a sense of improved communication when interacting with others who had a different perspective from his own:

*(P10N) “When I meet someone with an opinion different from my own, I take a moment and think. My instinct is less likely to be that their thoughts are wrong and more that they are different and that I may be able to find the common ground in between.”*

## Discussion

### Summary of Findings

This study aimed to explore (1) changes in understanding between autistic and non-autistic participants and (2) double empathy exchanges around empathising and perspective-taking, through the shared contemplation of serious literature. Relative findings are discussed below in relation to previous research and theory.

#### Literature as Risk Permitting

Data supported the argument that serious literature forces readers to “bite off more than they can chew,” promoting complex, open assessments of what was being read (Davis, 2020; Davis and Magee, 2020). This prevented participants from narrowing their understandings down into simplistic, stereotyped explanations of complex human experience (O’Sullivan et al., 2015; Davis and Magee, 2020). Although the non-autistic participants included in the study did not exhibit stigma toward autistic people in general or within their research pairs, all described having come to the study with some level of stereotyped views of autistic people that were subsequently challenged. This indicates a potential usefulness of literature in challenging these stereotyped views and possible associated stigma that exists toward autistic people (Cage et al., 2018; Pearson and Rose, 2021). While the lived experience of the autistic participants was reported as a key catalyst for these changes, it was the literature itself that prompted imaginative feeling within pairs, in present time. Similarly, although the shared experience of having both read the book was important in uniting pairs, the emotional atmosphere was deepened by the complex literary language within the book: the literary language, through its engagement with raw human emotions, turned the story into an emotionally complex, immersive environment for participants to operate within. In this way, participants went beyond simple discussions around disability and stigma prompted by the book, to operating more thoroughly within the text in a way that enabled them to feel together with the characters. This sharing in raw emotions resulted in an overcoming of the double empathy problem (Milton, 2012), enabling participants to feel for one another in the same way. Overall, this supports the idea that literature may be particularly provocative of empathic responses and subsequent perspective-taking (Koopman and Hakemulder, 2015; Davis and Magee, 2020).

Furthermore, the literature afforded a sense of safety for social explorations through individual risk taking. This resulted in disclosures of difficult past experiences as well as direct emotional text responses within pairs. This indicates that the current methodology may afford at least some of the benefits observed in shared reading groups (Longden et al., 2015), while also taking into account and ameliorating concerns autistic people may have about live shared readings (Chapple et al., 2021). Additionally, the autistic participants in this study reported concerns around being stereotyped, and consequently stigmatised, that led to some generalised social reluctance. However, the shared warmth and security afforded by the literature resulted in explorations of social difference within pairs. As a result, participants incorporated the duality of their interactions, rather than attributing blame for difficulties that occurred. This contrasts with everyday inter-neurotype communications, where stereotyping and social heuristics result in assumptions of autistic social deficits (Chown, 2014; Milton et al., 2018). This shared appreciation resulted in reports of autistic participants feeling that their differing views were validated by their partners. This further highlights the double empathy problem within everyday inter-neurotype interactions, where autistic people are often encouraged toward an assumed ideal of neuronormativity (Mueller, 2020). Furthermore, this demonstrates the value of shared reading in promoting a multiplicitous thinking style (Ida, 2020) that frames autistic people as having different and valued perspectives.

#### Literature as an Advantageous Double Empathy Methodology

Importantly, the inherent social nature of fiction that mirrors the complexity of real socio-emotional human experience (Zunshine, 2011; Mumper and Gerrig, 2019) resulted in pairs focussing on their shared, essential experience of human emotion, regardless of their categorical neurotype group. This indicates that literature may be advantageous in tackling the double empathy problem, by challenging problematic social assumptions stemming from “us and them” conceptualisations (Goffman, 1990; Cage et al., 2018; Pearson and Rose, 2021). This move from thinking in terms of categorical neurotype differences, toward thinking as readers and, on a wider scale, human beings shows that shared reading can achieve the dismissal of groupness argued necessary for maximal double empathy understandings (Ida, 2020). In this way, the double empathy problem was resolved amongst participants by transcending these norms and expectations to produce shared and effective communication. This supports Ida’s (2020) argument that in order to achieve double empathy and promote neurodiversity, there is a need for open, individualised assessments without binary conceptual framings.

Crucially for this study, the complexity of emotive understanding and response that is required by literature provided live evidence against assumptions that autistic people lack the emotional and social intelligence that is at the core of human experiences. Furthermore, responses to the disadvantaged Lennie fed off these powerful basic human feelings. This prompted participants to start feeling together with Lennie, who was felt as another human presence in the discussions. As a result, participants shared discussions about these core human experiences, adding to the socio-emotional complexity of the thinking. For example, engagement with the literature and characters resulted in conversations about various forms of stigma in wider society. This aligns with discussions that are regularly prompted through shared reading methodologies (Longden et al., 2015), again demonstrating that the current methodology may prompt parallel outcomes in a more comfortable way for autistic participants. Furthermore, it is these explorations of core human situations which are not readily experienced in general, everyday conversations. This rawness in exploring human experience, within a safe setting, encouraged slower assessments of social context, as opposed to the more (neuro)typical reliance on quick attributions. This renewed patience for careful social and individual exploration meant that participants reported intent to sensitively explore differing perspectives in the future, indicating that shared reading may prompt longer-term re-framings away from stereotyped understandings. This supports the important arguments of Ida (2020) and McCreadie and Milton (2020), that open and creative methodologies are needed to effectively overcome the double empathy problem.

#### Creative Overcoming Contesting Deficit Models

Participants demonstrated contrasting thoughts and feelings towardcharacters which were experienced in their complexity rather than being “resolved” into simplified conclusions. Given that all autistic participants demonstrated this overcoming, these findings challenge dominant theoretical framings of autism as being inherently associated with a reduced capacity for empathy and perspective-taking (Baron-Cohen, 1997, 2002, 2009). Furthermore, fictional contemplation, it is argued, requires higher-order empathy (Zunshine, 2011) that is furthered by shared communication around reading (Longden et al., 2015). The autistic participants here went beyond the ability to process the complex socio-emotional aspects of the text, but also added deeper levels of their own socio-emotional insight. This demonstration clearly conflicts with arguments that autistic individuals have inherent social and emotional impairments (Baron-Cohen, 2009; Lombardo and Baron-Cohen, 2011; Bodner et al., 2015; Rigby et al., 2018).

Where this overcoming was implemented during times of social difficulty within pairs, there resulted a sensitive understanding and move toward mutual resolution. Specifically, within all pairs, socio-communicative difficulties occurred due to autistic participants desiring structure, and non-autistic participants not wanting to over-dominate. As a result, these social difficulties did not lead to communication breakdowns, and subsequent blame attribution that is often associated with inter-neurotype communicative difficulties (Milton, 2012; Chown, 2014). Instead, participants took time and care to consider the problem, working together in building a social structure that worked for both. This transference of the slow and careful processing that the literature encouraged supports the view that the salience of literature results in contextual behavioural change (Mumper and Gerrig, 2019). Furthermore, this movement away from quick attributions of blame amidst communicative ambiguity implies a wider move away from deficit framings based on assumed general norms. This, together with feedback provided by participants after the study, further supports the idea that changes resulting from literary contemplations may result in wider changes in an individual’s social norms (Mumper and Gerrig, 2019).

### Limitations and Future Research

The willingness of the non-autistic participants to take part in research that was seeking to explore interactions with autistic participants indicates a pre-existing willingness to co-operatively engage with autistic people. Therefore, conclusions on how much the literature brought about a change in understandings are limited to this sample. Additionally, the participants in this study were willing to read and discuss literature, and so may have been more readily willing to engage with reflexive thinking than most. For people with pre-existing stigmatising views about autism and autistic people, it remains a question as to whether the shared reading paradigm used here would be ethically and socially appropriate. Future research should seek to explore whether literature that has a neurodiversity focus would bring about double empathy understandings for non-autistic people while reading alone. This is important in order to explore how reading can be used as a double empathy intervention tool for individuals who hold particularly stigmatising views toward autistic people.

Additionally, the methodology implemented in this research lacks the text liveness that is important in other shared reading designs, such as reading aloud groups (Longden et al., 2015; Davis, 2020). Therefore, more research is needed to explore text liveness within shared readings between autistic and non-autistic people in a way that remains comfortable. For example, expansion of the current methodological design could seek to explore the added value of having participants select and read aloud passages which move them. It is also important to identify how larger-scale or longer-term shared reading paradigms might be designed and implemented, given concerns that book club style groups may result in limited demographic inclusion (Davis and Magee, 2020). While this study indicated that the shared experience specific to literature promoted deeper discussions, future research should seek to compare shared reading with discussions of other shared experiences.

The sample used here is also limited because autistic adults were only included if they did not have an additional disability that would affect their reading and writing skills. Similarly, all autistic participants in this study communicated verbally, resulting in limited representation of the autistic community. As a result, more research is needed to assess the utility of shared reading as a means to overcome the double empathy problem where individuals have additional support needs.

## Conclusion

In conclusion, the findings of this study show the potential utility of serious literature for overcoming the double empathy problem (Milton, 2012). Importantly, the literature resulted in a focus on overarching, essential human similarities, even through felt differences. This moved participants away from binary group assessments that often result in stereotyping and subsequent stigma within general society (Cage et al., 2018; Pearson and Rose, 2021). Therefore, findings imply that shared reading promotes multiplicity (Ida, 2020), moving participants toward a shared identity with sensitive considerations of difference. Importantly, findings contest dominant deficit-based theories of autism (Baron-Cohen, 1997, 2002, 2009), showing that autistic people do empathically respond to the perspectives of others. Similarly, these findings of autistic people engaging emotionally with serious literature contest over-simplistic framings of autistic individuals as inherently lacking in social and emotional understanding (Baron-Cohen, 2009; Lombardo and Baron-Cohen, 2011). In this study, all participants showed the higher-order levels of empathising and perspective-taking necessary for fictional contemplation (Zunshine, 2011). Overall, the findings here support arguments that open, creative research methodologies, fostering a broader shared understanding, are useful for achieving effective double empathy understandings (McCreadie and Milton, 2020; Mueller, 2020). As Steinbeck (1952, p. 444) himself wrote:

*“You can only understand people if you feel them in yourself.”*

## Data Availability Statement

The datasets presented in this article are not readily available because the ethical approval obtained from the University of Liverpool covered the publication of unrecognisable sections of anonymised data only. Due to the sensitive nature of the data, anonymised data cannot be provided in full due to the potential for participants to be recognised. Requests to access the datasets should be directed to MC.

## Ethics Statement

The studies involving human participants were reviewed and approved by the Health and Life Sciences Research Ethics Committee (Psychology, Health and Society), University of Liverpool. The patients/participants provided their written informed consent to participate in this study.

## Author Contributions

MC, PD, JB, and RC: conceptualization, methodology, and validation. MC: funding acquisition, data curation, project administration, and writing – original draft. MC, PD, JB, JAM, CR, and RC: formal analysis, writing, review, and editing. PD, JB, and RC: supervision. All authors contributed to the article and approved the submitted version.

## Conflict of Interest

The authors declare that the research was conducted in the absence of any commercial or financial relationships that could be construed as a potential conflict of interest.

## Publisher’s Note

All claims expressed in this article are solely those of the authors and do not necessarily represent those of their affiliated organizations, or those of the publisher, the editors and the reviewers. Any product that may be evaluated in this article, or claim that may be made by its manufacturer, is not guaranteed or endorsed by the publisher.
